# Supplementary material for: Children’s risk preferences vary across sexes, social contexts, and cultures
Source: Commun Psychol. 2024 Aug 23;2:79. doi: 10.1038/s44271-024-00127-z (PMC11343856; doi:10.1038/s44271-024-00127-z)
Supplement: Supplementary file 2 — Supplementary Information [file 44271_2024_127_MOESM2_ESM.pdf]

Children's Risk Preferences Vary Across Sexes, Social Contexts, and Cultures

–Supplementary Information–

Table of contents

Notes

Supplementary Note 1: Study Devices and Materials

Supplementary Note 2: First Trial Analyses

Supplementary Note 3: Codebook

Supplementary Note 4: Full Data Set Analyses (Revision)

Supplementary Note 5: Trial Order Effects (Revision)

Supplementary Note 6: Test Role and Order Effects (Revision)

Supplementary Note 7: Full Model Parameters (Estimates and Standard Errors; Revision)

Figures

Supplementary Figure S1: Study Devices and Materials

Supplementary Figure S2: Risk Preferences depending on sex, condition, and culture (First Trial)

Supplementary Figure S3: Risk preferences across ages, conditions, and cultures (First Trial)

Supplementary Figure S4: Trial order effects

Tables

Supplementary Table S1. Codebook

Supplementary Table S2. Full Model Parameters

Data and Code are available via the Open Science Framework (https://osf.io/3nukt/).

Supplementary Note 1: Study Devices and Materials

Supplementary Figure S1: Study Devices and Materials

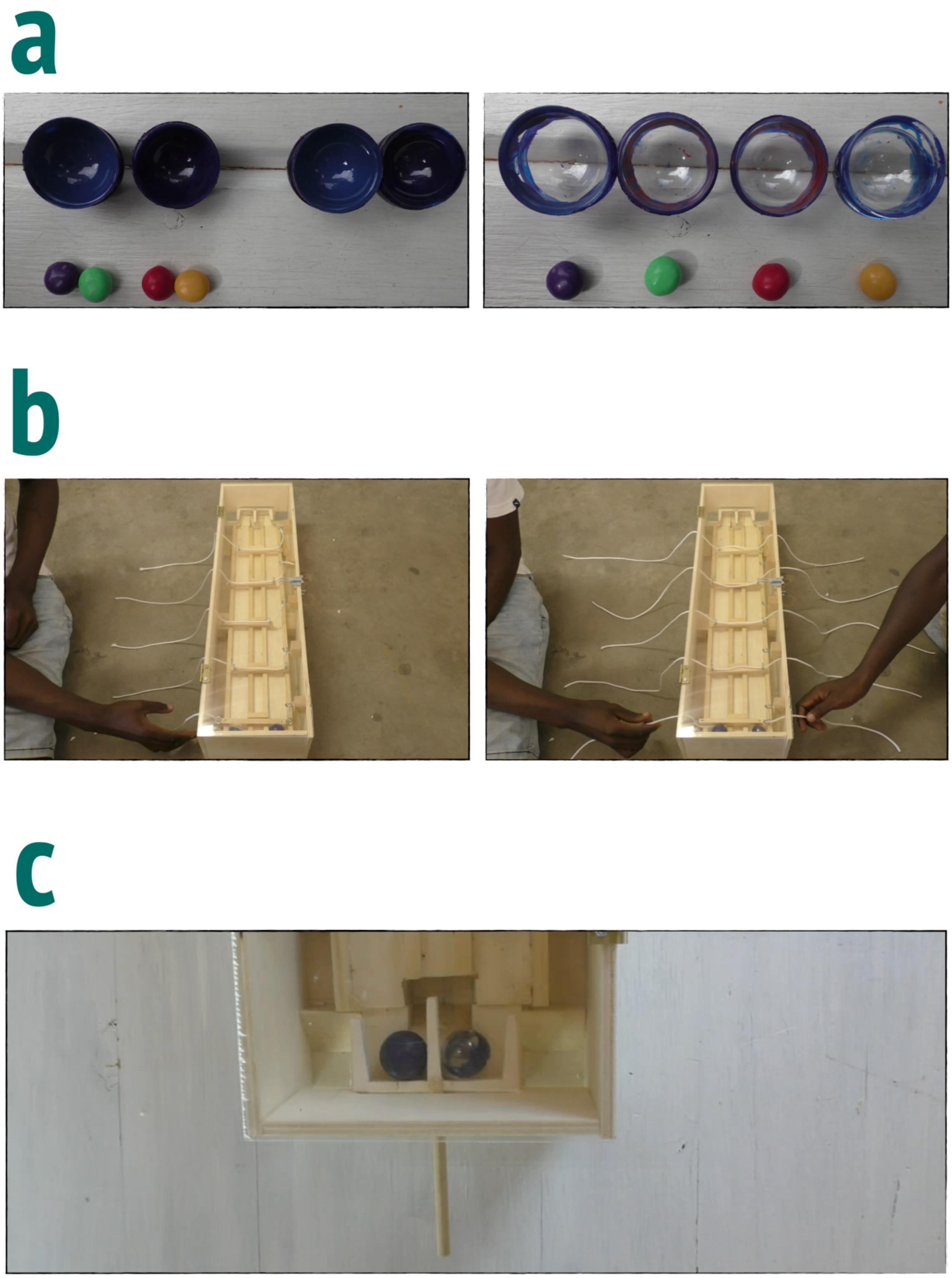

Supplementary Figure S1: Study devices and materials; (A) risky (left) and safe (right) options as presented to children; (B) moving the safe and risky options down the ramp individually (left) and collaboratively (right); (C) handle to choose between risky (left) and safe (right) option

Supplementary Note 2: First Trial Analyses

In this section, we provide additional information on a first trial analysis in which we focus on children's risk preferences exclusively in the first (counterbalanced) condition they encountered in the study. We did so to remove potential carry-over effects between conditions as children engaged in different social settings as part of a within-subjects research design. Naturally, this approach leads to increased uncertainty in estimated probabilities due to data loss.

Risk Preferences: General Patterns (First Trial)

In the individual condition, both Hai||om ($\text{probability}_{Boys}$ [95%-HPD] = 0.34 [0.22; 0.46], $\text{probability}_{Girls}$ [95%-HPD] = 0.32 [0.21; 0.44]) and Ovambo children ($\text{probability}_{Boys}$ [95%-HPD] = 0.37 [0.26; 0.49], $\text{probability}_{Girls}$ [95%-HPD] = 0.35 [0.24; 0.47]) showed risk aversion. A similar tendency was observed in the observation condition, although 95%-HPD intervals encompassed chance level at probability = .50 (Hai||om: $\text{probability}_{Boys}$ [95%-HPD] = 0.45 [0.32; 0.59], $\text{probability}_{Girls}$ [95%-HPD] = 0.35 [0.21; 0.51]; Ovambo: $\text{probability}_{Boys}$ [95%-HPD] = 0.50 [0.36; 0.64], $\text{probability}_{Girls}$ [95%-HPD] = 0.37 [0.24; 0.51]). For the collaboration condition, most groups of children also exhibited risk aversion (Hai||om: $\text{probability}_{Boys}$ [95%-HPD] = 0.27 [0.16; 0.40], $\text{probability}_{Girls}$ [95%-HPD] = 0.19 [0.09; 0.32]; Ovambo: $\text{probability}_{Boys}$ [95%-HPD] = 0.43 [0.30; 0.58], $\text{probability}_{Girls}$ [95%-HPD] = 0.31 [0.19; 0.45]).

Risk Preferences Across Sexes, Conditions, and Cultures (First Trial)

Pairwise contrasts suggested that boy' risk preferences were stronger in the observation condition compared to when being alone (Observation > Individual: $\text{contrast}_{\text{Hai||}om}$ = .92, $\text{contrast}_{\text{Ovambo}}$ = .93). Among the Ovambo, this pattern was somewhat weaker for the collaboration condition (Collaboration > Individual: $\text{contrast}_{\text{Ovambo}}$ = .75), whereas Hai||om boys tended to seek more risks in individual settings compared to collaboration ($\text{contrast}_{\text{Hai||}om}$ = .20). Thus, boys sought less risks following collaboration compared to the observation condition (Collaboration > Observation: $\text{contrast}_{\text{Hai||}om}$ = .02, $\text{contrast}_{\text{Ovambo}}$ = .25). In the first condition alone, girls' risk preferences were robust to peer presence (Observation > Individual:

$contrast_{Hai||om}$ = .63, $contrast_{Ovambo}$ = .59). However, girls from both cultures appear averse to risks during collaboration than the other two conditions (Collaboration > Individual: $contrast_{Hai||om}$ = .04, $contrast_{Ovambo}$ = .32; Collaboration > Observation: $contrast_{Hai||om}$ = .04, $contrast_{Ovambo}$ = .27). In sum, these results suggest that Hai||om children were particularly averse to risks following peer collaboration.

At the beginning of the study, both Hai||om (Boys > Girls: $contrast_{Observation}$ = .86; $contrast_{Collaboration}$ = .85) and Ovambo girls ($contrast_{Observation}$ = .92; $contrast_{Collaboration}$ = .91) were more risk averse than boys when accompanied by their peers. However, such an effect was much less pronounced for individual settings, were we found no credible evidence that Hai||om ($contrast_{Individual}$ = .59) and Ovambo ($contrast_{Individual}$ = .61) boys would more aprone to risks than girls in the respective community.

Finally, as suggested by the main analyses, Ovambo children were less risk averse than their Hai||om counterparts. This effect was evident both for boys (Ovambo > Hai||om: $contrast_{Individual}$ = .70; $contrast_{Observation}$ = .69; $contrast_{Collaboration}$ = .97) and for girls ($contrast_{Individual}$ = .67; $contrast_{Observation}$ = .59; $contrast_{Collaboration}$ = .94). Note that contrasts were highest for the collaboration condition. Children's risk preferences across social contexts, sexes, and cultures in the initial phase of the study are visualized in Figure S2.

Supplementary Figure S2: Risk Preferences depending on sex, condition, and culture (First Trial)

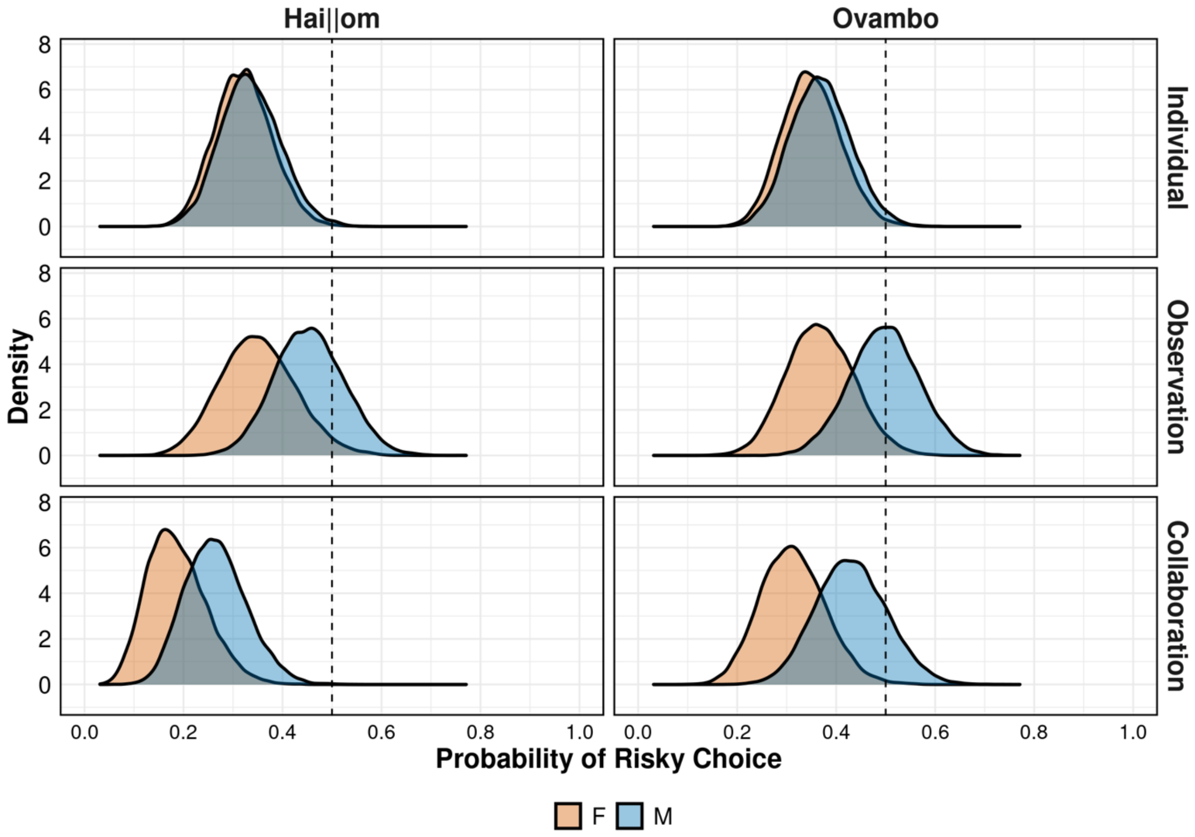

Supplementary Figure S2. Posterior probabilities illustrating the estimated probability that a child will choose the risky option depending on their sex, condition, and culture (first condition tested per child). Densities are based on the full model to illustrate all potential interactions between predictors. Children's age is set at the mean. Dotted vertical lines represent chance level at $\text{probability}_{\text{Chance}} = .50$

Risk Preferences: Developmental Dynamics (First Trial)

To further shed light on developmental variation in the effects of condition, sex, and culture on children's risk preferences, we estimated contrasts of the mean posterior probabilities for children's risk preferences at the older and younger age limits tested. These contrasts indicated a trend towards increased risk aversion among older Hai||om boys (Older – Younger: $\text{contrast}_{\text{Individual}} = -.27$; $\text{contrast}_{\text{Observation}} = -.18$; $\text{contrast}_{\text{Collaboration}} = -.12$; again, contrasts of .00

indicate developmental stability). A similar trend was observed among Hai||om girls ($contrast_{Individual}$ = -.20; $contrast_{Collaboration}$ = -.18; but $contrast_{Observation}$ = .04). For Ovambo children, we found no credible evidence for developmental variation in risk preferences among boys ($contrast_{Individual}$ = .05; $contrast_{Observation}$ = -.03; $contrast_{Collaboration}$ = .18) or girls ($contrast_{Individual}$ = .09; $contrast_{Observation}$ = .15; but $contrast_{Collaboration}$ = .01). Age-related trajectories are visualized in Figure S3.

Supplementary Figure S 3: Risk preferences across ages, conditions, and cultures (First Trial)

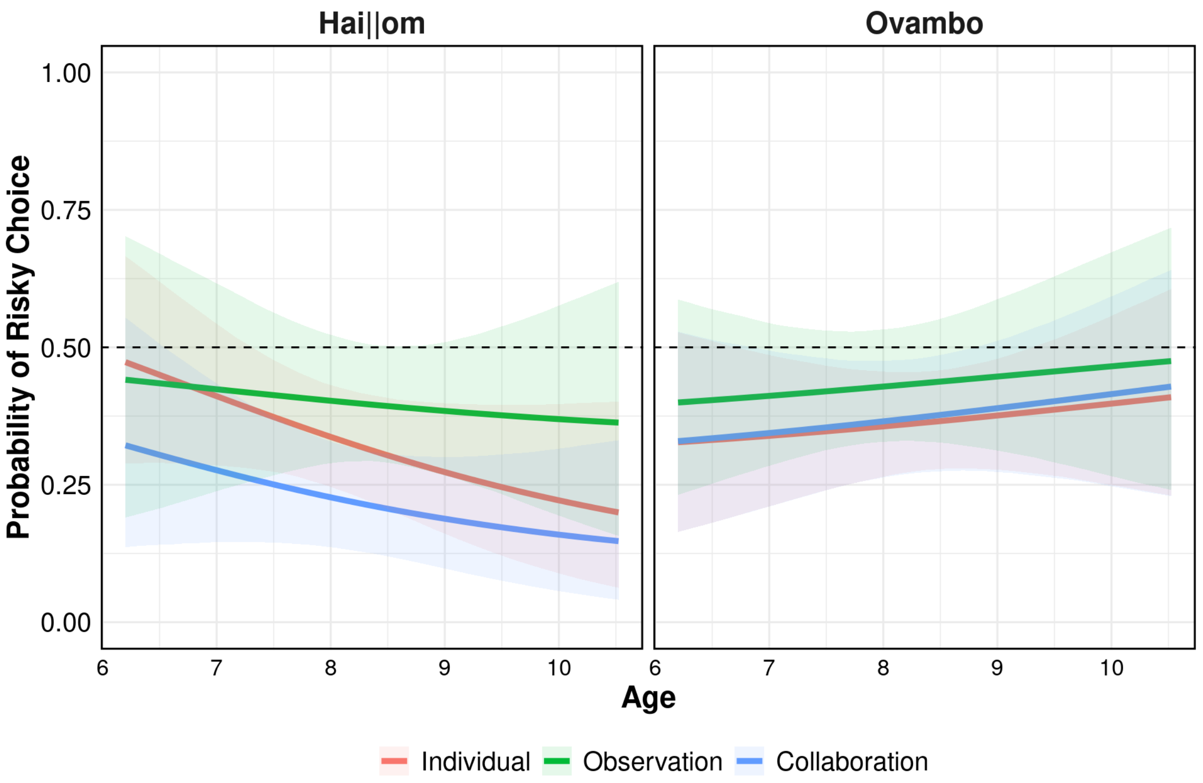

Supplementary Figure S3. Posterior probabilities illustrating the estimated probability that a child will choose the risky option depending on their age, condition, and culture. The first condition in which children participated is plotted. Densities are based on the full model to illustrate all potential interactions between predictors. Children's sex is centered at zero. Solid lines present posterior means, surrounding areas present 95%-HPDs. Dotted horizontal lines represent chance level at $probability_{Chance}$ = .50.

Supplementary Note 3: Codebook ("Supplementary Data 1.csv")

Variable	Type	Values	Further Information
ID	factor	[1 - 144]	participant ID
RISK	integer	[0; 1]	risk preference in a given trial<br>(0 = Safe Choice; 1 = Risky Choice)
CONDITION	Factor	[I; O; C]	condition of a given trial (assessed within-subjects)<br>(I = Individual; O = Observation; C = Collaboration)
z.CULTURE	numeric	[-0.5; 0.5]	cultural context<br>(-0.5 = Hai||om; 0.5 = Ovambo)
z.SEX	numeric	[-0.5; 0.5]	child's sex<br>(-0.5 = male; 0.5 = female)
z.AGE	numeric	[-1.74 - 1.95]	child's age (z-scaled)
z.SIDE	numeric	[-0.5; 0.5]	location of risky option from child's point of view<br>(-0.5 = left; 0.5 = right)
FIRST	factor	[T; F]	first condition assessed for a given child<br>(T: first condition; F: second or third condition)

Supplementary Table S1. Codebook

Supplementary Note 4: Full Data Set Analyses (Revision)

Here, we report the results of an analysis including the additional n = 19 children who showed exclusive side biases throughout the study. All other aspects of the analyses remain identical to the analyses reported in the manuscript. As shown below, including these children does not affect the main conclusions drawn from the study.

Risk Preferences: General Patterns (Full Data Set)

Both Hai||om (probability$_{Boys}$ [95%-HPD] = 0.27 [0.18; 0.38], probability$_{Girls}$ [95%-HPD] = 0.20 [0.13; 0.30]) and Ovambo children (probability$_{Boys}$ [95%-HPD] = 0.31 [0.21; 0.43], probability$_{Girls}$ [95%-HPD] = 0.26 [0.18; 0.37]) showed risk aversion when tested individually. This tendency was also evident in the observation condition (Hai||om: probability$_{Boys}$ [95%-HPD] = 0.38 [0.26; 0.51], probability$_{Girls}$ [95%-HPD] = 0.22 [0.13; 0.33]; Ovambo: probability$_{Boys}$ [95%-HPD] = 0.38 [0.26; 0.51], probability$_{Girls}$ [95%-HPD] = 0.30 [0.20; 0.41]). and the collaboration condition (Hai||om: probability$_{Boys}$ [95%-HPD] = 0.32 [0.21; 0.45], probability$_{Girls}$ [95%-HPD] = 0.17 [0.09; 0.26]; Ovambo: probability$_{Boys}$ [95%-HPD] = 0.41 [0.29; 0.54], probability$_{Girls}$ [95%-HPD] = 0.31 [0.21; 0.44]).

Risk Preferences Across Sexes, Conditions, and Cultures (Full Data Set)

Boy' risk preferences were stronger in the observation condition compared to the individual condition (Observation > Individual: contrast$_{\text{Hai||}om}$ = .98, contrast$_{\text{Ovambo}}$ = .88). The same pattern emerged for the collaboration condition (Collaboration > Individual: contrast$_{\text{Hai||om}}$ = .80; Collaboration > Individual: contrast$_{\text{Ovambo}}$ = .96). Overall, there was no clear tendency of boys seeking more or less risks following collaboration compared to the observation condition (Collaboration > Observation: contrast$_{\text{Hai||}om}$ = .16, contrast$_{\text{Ovambo}}$ = .71).

Ovambo girls' risk tended to take more risks during peer presence compared to individual settings (Observation > Individual: contrast$_{\text{Ovambo}}$ = .77; Collaboration > Individual: contrast$_{\text{Ovambo}}$ = .83; Collaboration > Observation: contrast$_{\text{Ovambo}}$ = .58), although this tendency was less pronounced compared to their boy peers. For Hai||om girls, pairwise contrasts did not

indicate such an effect (Observation > Individual: $\text{contrast}_{\text{Hai||om}}$ = .64; Collaboration > Individual: $\text{contrast}_{\text{Hai||om}}$ = .17; Collaboration > Observation: $\text{contrast}_{\text{Hai||om}}$ = .12).

Across conditions, girls were more risk averse than boys among the Hai||om (Boys > Girls: $\text{contrast}_{\text{Individual}}$ = .88; $\text{contrast}_{\text{Observation}}$ = .98; $\text{contrast}_{\text{Collaboration}}$ = .99) and the Ovambo (Boys > Girls: $\text{contrast}_{\text{Individual}}$ = .78; $\text{contrast}_{\text{Observation}}$ = .84; $\text{contrast}_{\text{Collaboration}}$ = .89). Note that, however, these differences were markedly stronger during peer presence.

Ovambo children appeared less averse to risks than their Hai||om counterparts. For girls, this tendency was evident across conditions (Ovambo > Hai||om: $\text{contrast}_{\text{Individual}}$ = .86; $\text{contrast}_{\text{Observation}}$ = .89; $\text{contrast}_{\text{Collaboration}}$ = .99). For boys, a similar pattern emerged in the individual condition ($\text{contrast}_{\text{Individual}}$ = .74) and the collaboration condition ($\text{contrast}_{\text{Collaboration}}$ = .87). Only when observed by a peer, Hai||om and Ovambo boys showed similar risk preferences ($\text{contrast}_{\text{Observation}}$ = .49).

Supplementary Note 5: Trial Order Effects (Revision)

To test whether children's risk preferences varied across trials, we fitted a simple monotonic model in brms with the predictor "trial" on children's risk preferences. This analysis does not reveal any evidence for such an effect (mo_{Trial} (95%-CI) = -0.01 (-0.04; 0.02)). Estimated trial effects are plotted below together with mean probabilities for risk-seeking behaviors.

Supplementary Figure S4: Trial order effects

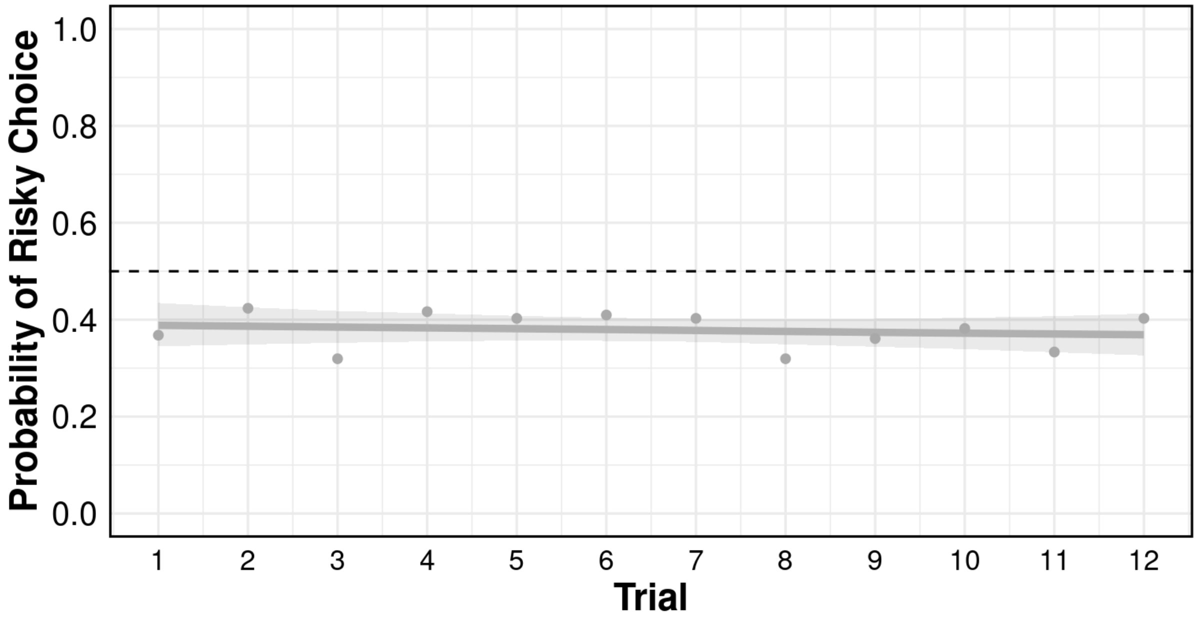

Supplementary Figure S4. Estimated monotonic effect of trial order on risk preferences. Dotted line indicates chance level at prob = 0.5; solid line represents model estimates; grey areas indicate 95%-HPD intervals; dots represent mean probabilities per trial

Supplementary Note 6: Test Role and Order Analyses (Revision)

Finally, we tested whether children who participated only as target children (n = 26; $n_{Hai||om}$ = 13, 6 girls; n_{Ovambo} = 13, 4 girls) showed different risk preferences from their peers who participated in both roles at some point. Note that all children were asked to participate as confederates if they were available at the study location following their participation. We fitted a simple Bayesian regression model with Bernoulli response distribution, predicting children's risk preferences with a dichotomous variable ("One Task Only"). This analysis does not reveal any evidence for such an effect (Estimate (95%-CI) = -0.09 (-0.36; 0.18); $\text{probability}_{Target\ Only}$ = *.34,* $\text{probability}_{Both\ Roles}$= *.36*).

Also, we investigated whether children who participated as confederates first (n = 7; $n_{Hai||om}$ = 4, 3 girls; n_{Ovambo} = 3, 1 girl)) differed from their peers who participated as target children first. Note that the former group of children participated early in the study phases at each study location to get data collection started. We fitted a Bayesian regression model with Bernoulli response distribution, predicting risk preferences with a dichotomous variable ("Confederate as First Role"). This analysis does not reveal evidence for such an effect (Estimate (95%-CI) = -0.27 (-0.82; 0.24); $\text{probability}_{Confederate\ First}$ = *.31,* $\text{probability}_{Confederate\ Second}$ = *.36*).

Supplementary Note 7: Full Model Parameters

	Estimate	Est. Error	l-95% CI	u-95% CI
**Group-Level Effects**				
~ID				
sd (Intercept)	1.22	0.12	0.99	1.47
sd (z.Side)	1.05	0.19	0.67	1.42
Cor (Intercept, z.Side)	-0.25	0.19	-0.61	0.13
**Population-Level Effects**				
Condition: Individual	-1.03	0.16	-1.36	-0.72
Condition: Observation	-0.77	0.16	-1.09	-0.45
Condition: Collaboration	-0.89	0.17	-1.22	-0.57
z.Sex	-0.34	0.28	-0.90	0.21
z.Culture	0.30	0.27	-0.17	0.85
z.Age	0.05	0.14	-0.23	0.34
Condition_Observation*z.Sex	-0.25	0.26	-0.81	0.22
Condition_Collaboration*z.Sex	-0.37	0.28	-0.96	0.12
Condition_Observation*z.Culture	-0.06	0.24	-0.56	0.41
Condition_Collaboration*z. Culture	0.38	0.28	-0.12	0.96
z.Sex*z.Culture	0.19	0.39	-0.53	1.04
Condition_Observation*z.Age	-0.00	0.14	-0.27	0.27
Condition_Collaboration*z.Age	-0.05	0.14	-0.34	0.22
z.Sex*z.Age	-0.15	0.24	-0.67	0.30
z.Culture*z.Age	0.40	0.28	-0.10	0.97
Condition_Observation*z.Sex*z.Culture	0.31	0.41	-0.41	1.20
Condition_Collaboration*z.Sex*z.Culture	0.36	0.43	-0.38	1.30
Condition_Observation*z.Sex*z.Age	0.18	0.25	-0.29	0.70

Condition_Collaboration*z.Sex*z.Age	0.16	0.25	-0.30	0.69
Condition_Observation*z.Culture *z.Age	-0.23	0.26	-0.78	0.24
Condition_Collaboration*z.Culture *z.Age	-0.13	0.25	-0.66	0.34
z.Sex*z.Culture*z.Age	0.17	0.39	-0.55	1.03
Condition_Observation*z.Sex*z.Culture*z.Age	0.06	0.38	-0.70	0.86
Condition_Collaboration*z.Sex*z.Culture*z.Age	-0.13	0.39	-0.97	0.63

Supplementary Table S2. Full Model Parameters
