# Supplementary material for: Children’s risk preferences vary across sexes, social contexts, and cultures
Source: Commun Psychol. 2024 Aug 23;2:79. doi: 10.1038/s44271-024-00127-z (PMC11343856; doi:10.1038/s44271-024-00127-z)
Supplement: Supplementary file 3 — Description of Additional Supplementary Files [file 44271_2024_127_MOESM3_ESM.pdf]

Description of Additional Supplementary Files

File name: Supplementary Data 1

File description: Dataset to reproduce analyses.
