# Supplementary material for: Children’s risk preferences vary across sexes, social contexts, and cultures
Source: Commun Psychol. 2024 Aug 23;2:79. doi: 10.1038/s44271-024-00127-z (PMC11343856; doi:10.1038/s44271-024-00127-z)
Supplement: Supplementary file 5 — Reporting Summary [file 44271_2024_127_MOESM5_ESM.pdf]

nature portfolio

Corresponding author(s): Roman Stengelin

Last updated by author(s): May 21, 2024

Reporting Summary

Nature Portfolio wishes to improve the reproducibility of the work that we publish. This form provides structure for consistency and transparency in reporting. For further information on Nature Portfolio policies, see our Editorial Policies and the Editorial Policy Checklist.

Statistics

For all statistical analyses, confirm that the following items are present in the figure legend, table legend, main text, or Methods section.

n/a	Confirmed	
☐	☒	The exact sample size (*n*) for each experimental group/condition, given as a discrete number and unit of measurement
☐	☒	A statement on whether measurements were taken from distinct samples or whether the same sample was measured repeatedly
☐	☒	The statistical test(s) used AND whether they are one- or two-sided<br>*Only common tests should be described solely by name; describe more complex techniques in the Methods section.*
☐	☒	A description of all covariates tested
☒	☐	A description of any assumptions or corrections, such as tests of normality and adjustment for multiple comparisons
☐	☒	A full description of the statistical parameters including central tendency (e.g. means) or other basic estimates (e.g. regression coefficient) AND variation (e.g. standard deviation) or associated estimates of uncertainty (e.g. confidence intervals)
☒	☐	For null hypothesis testing, the test statistic (e.g. *F*, *t*, *r*) with confidence intervals, effect sizes, degrees of freedom and *P* value noted<br>*Give P values as exact values whenever suitable.*
☐	☒	For Bayesian analysis, information on the choice of priors and Markov chain Monte Carlo settings
☐	☒	For hierarchical and complex designs, identification of the appropriate level for tests and full reporting of outcomes
☐	☒	Estimates of effect sizes (e.g. Cohen's *d*, Pearson's *r*), indicating how they were calculated

Our web collection on statistics for biologists contains articles on many of the points above.

Software and code

Policy information about availability of computer code

Data collection: NA

Data analysis: All statistical analyses were conducted in R (R Core Team, 2022; version 4.3.1). The code for all analyses has been made publicly available.

For manuscripts utilizing custom algorithms or software that are central to the research but not yet described in published literature, software must be made available to editors and reviewers. We strongly encourage code deposition in a community repository (e.g. GitHub). See the Nature Portfolio guidelines for submitting code & software for further information.

Data

Policy information about availability of data

All manuscripts must include a data availability statement. This statement should provide the following information, where applicable:

- Accession codes, unique identifiers, or web links for publicly available datasets
- A description of any restrictions on data availability
- For clinical datasets or third party data, please ensure that the statement adheres to our policy

Data and code have been made publicly available at the Open Science Framework.

Research involving human participants, their data, or biological material

Policy information about studies with human participants or human data. See also policy information about sex, gender (identity/presentation), and sexual orientation and race, ethnicity and racism.

Reporting on sex and gender	We refer to sex as a biological category throughout the manuscript as we recruited participants from educational institutions and their households relying on the sex assigned to children at birth. We do so in accordance with prior research on risk preferences in small-scale societies. We did not gather separate data on gender.<br>Based on our approach it is impossible to dissect effects of sex and gender on children's risk preferences.
Reporting on race, ethnicity, or other socially relevant groupings	We grouped children based on the communities in which they were recruited, the languages spoken at their homes, and their caregivers' ethnicities.
Population characteristics	See above (and ethnographic information and study rationale provided in the manuscript)
Recruitment	Children were recruited through an opportunity sampling approach. All available children participated in the research.
Ethics oversight	Ethics Committee at Leipzig University & WIMSA

Note that full information on the approval of the study protocol must also be provided in the manuscript.

Field-specific reporting

Please select the one below that is the best fit for your research. If you are not sure, read the appropriate sections before making your selection.

☐ Life sciences ☒ Behavioural & social sciences ☐ Ecological, evolutionary & environmental sciences

For a reference copy of the document with all sections, see nature.com/documents/nr-reporting-summary-flat.pdf

Behavioural & social sciences study design

All studies must disclose on these points even when the disclosure is negative.

Study description	The study type is a quantitative experiment with 3 conditions being assessed within-subjects. Children from two cultural contexts were assessed as part of a cross-cultural study.
Research sample	Children from two traditional indigenous Namibian communities were assessed: A Hai||om community (traditionally foragers) and an Ovambo community (traditionally agro-pastoralists, today often engaging in the national economy). In each community, 72 children aged 6-10 years were included in the study, with equal numbers of boys and girls at each site (36). Information on children's sex was obtained from their birth certificates and caregivers, while data on children's ethnicity was gathered from school teachers and caregivers. We relied on a opportunity-sampling approach but sampled almost all children available in the small communities and thus argue that these participants are highly representative of the local communities.
Sampling strategy	All eligible children in the communities were approached pending children's (and caregivers') consent. Minimum sample sizes were oriented on prior research in these communities and preregistered accordingly. Statistical analyses further account for uncertainty regarding statistical power.<br>Children were assigned randomly to condition sequences.
Data collection	Data was captured from video. The experimenter was in the room throughout the study and aware of hypotheses and condition but unaware of the content of the risky options. The experimenter moved to an adjacent room during children's decisions to avoid biasing their choices.
Timing	Data was obtained between January and April 2019.
Data exclusions	No children were excluded from the study.
Non-participation	No children declined participation in the study.
Randomization	Participants were randomly assigned to conditions.

Reporting for specific materials, systems and methods

We require information from authors about some types of materials, experimental systems and methods used in many studies. Here, indicate whether each material, system or method listed is relevant to your study. If you are not sure if a list item applies to your research, read the appropriate section before selecting a response.

Materials & experimental systems

n/a	Involved in the study
☒	☐ Antibodies
☒	☐ Eukaryotic cell lines
☒	☐ Palaeontology and archaeology
☒	☐ Animals and other organisms
☒	☐ Clinical data
☒	☐ Dual use research of concern
☒	☐ Plants

Methods

n/a	Involved in the study
☒	☐ ChIP-seq
☒	☐ Flow cytometry
☒	☐ MRI-based neuroimaging
